# A Case of Spontaneous Restoration of the Retroverted Gravid Uterus

**Published:** 1856-02

**Authors:** Francis H. Ramsbotham


### RECORD OF MEDICAL SCIENCE.

*A Case of Spontaneous Restoration of the Retroverted Gravid Uterus.* By FRANCIS H. RAMSBOTHAM, M. D.

In the *Medical Times and Gazette* for October 23, 1852, will be found eight cases of retroversion of the gravid uterus, treated by me, which go far to prove the truth of the position laid down by Denman,—in opposition to the doctrine inculcated by William Hunter, Baudelocque, and others,—that if the bladder be kept duly empty, the womb will, in the great majority of instances, spontaneously regain its natural position, without the employment of any manual operation, beyond the frequent introduction of the catheter.

Since some members of the Profession, however, may still incline to the opinion that the use of active means is absolutely required for its restoration, and, as consequently forcible attempts to push the fundus upwards, may very likely be had recourse to unnecessarily, I think the publication of every case that tends to confirm our reliance on the passive mode of treatment, useful; and with this view I beg to call attention to the following:—

A. G. of Hannibal Road, Stepney, aged forty-one years, married twenty-one years, having had nine children, and suffored two miscarriages,—the last at two months, in January of this year,—applied to me among the out-patients at the London Hospital, on Saturday, the 29th of last Septemper. She stated that she had not menstruated since the middle of June; that seven days before, while lifting a heavy pail full of water out of the water-butt, she felt a sudden pain at the lower part of the abdomen; this was followed by some difficulty in passing urine, though it did not amount to an absolute stoppage. On the next Tuesday however, after riding from Stepney to Pimlico in an omnibus, which shook her exceedingly, she found she was quite unable to expel the least quantity from the bladder. On her return home, the urine was drawn off by the catheter, and the same instrument was used again on Wednesday, but not by the same gentleman. From that day till I saw her on the Saturday, the catheter had not again been had recourse to, nor had she passed any urine voluntarily; though some had dribbled away at different times, or come in gushes. The bowels had not acted since Tuesday. She did not know whether she was pregnant or not; but inclined to the latter opinion.

She appeared to be in great pain, and her countenance was indicative of much distress. Independently of the agony of a distended bladder, she complained of a violent forcing and bearing down, and a tearing and dragging sensation at the groins.

The history and symptoms led me immediately to suspect that the uterus was gravid and retroverted. I found the bladder largely filling the lower half of the abdominal cavity, its fundus rising as high as the umbilicus; introduced the catheter without delay, and drew off the enormous quantity of ninety-two ounces of dark colored, but not offensive urine. On making a careful examination, my suspicions were confirmed; for the hollow of the sacrum was occupied by a hard tumor, which was, indeed, the fundus and body of the uterus; while the mouth of that organ was canted upwards and forwards, behind or rather above the symphysis pubis. The anterior face of the vagina was considerably stretched, and there was general tenderness of the whole pelvic structures.

I admitted her into the Hospital, and ordered her to bed; fomentations were applied, and she took 30 minims of laudanum. My resident assistant was directed to draw off the urine every eight hours, and to administer some castor-oil the next morning. The medicine acted satisfactorily on the bowels in the course of the day; the bladder was kept empty as described; she was confined scrupulously to the recumbent position; no attempt was made to replace the uterus artificially; nor was any fresh vaginal examination even instituted; and in three days she was able to void her urine of her own accord satisfactorily. The anxiety of countenance had then disappeared, and she expressed herself as feeling quite well. On examination per vaginam, the fundus uteri was now no longer to be felt in the pelvic cavity, the os uteri was in its natural situation, and none of the previous tenderness remained. I kept her in the Hospital merely for the sake of precaution, to prevent a recurrence of the accident, for a fortnight. During this interval she was confined to bed; was enjoined to pass water frequently; and, when she attempted to do so, to place herself on her hands and knees. She left the house on October 13, and is going on in pregnancy, not having experienced any return of the symptoms. She tells me that she quickened last week.

No case could more strongly prove the value of rest and the recumbent posture, together with a diligent attention to the bladder, under such circumstances, than this does. I scarcely expected that the uterus would have righted itself so speedily, after having been three days partially, and four completely, retroverted, with its fundus pressed so low down towards the perinæum as this was; and I was prepared to use some mechanical efforts to restore it, if it had continued in this abnormal position for two or three days longer.—*Medical Times and Gazette.*

